# Ambulatory models for autologous stem-cell transplantation: a systematic review of the health impact

**DOI:** 10.3389/fimmu.2024.1419186

**Published:** 2024-07-16

**Authors:** Miguel Ángel Prieto del Prado, Francesc Fernández Avilés

**Affiliations:** ^1^ Hematology Department, Hospital Universitario de Melilla, Melilla, Spain; ^2^ Hematology Department, Bone Marrow Transplantation Unit, Instituto del Cáncer y Enfermedades de la Sangre (ICAMS), Hospital Clínic, Barcelona, Spain

**Keywords:** autologous hematopoietic stem-cell transplantation, outpatient transplantation, quality of life, hospital-at-home, ambulatory

## Abstract

Autologous stem-cell transplantation (ASCT) is the standard of care for the management of multiple myeloma and has a well-established role in the treatment of some types of lymphoma. Over the last decades, the number of ASCT performed has increased significantly, leading to elevated pressure and cost for healthcare services. Conventional model of ASCT includes the admission of patients to a specialized Transplant Unit at any stage of the procedure. To optimize healthcare provision, ambulatory (outpatient/at-home) setting should be the focus moving forward. Thus, ambulatory ASCT model permits reducing average hospital stays and pressures on healthcare services, with significant cost-saving benefits and high degree of patient and caregiver satisfaction. In addition, it facilitates the bed resource for other complex procedures such as allografts or CAR-T cell therapy. The aim of this systematic review is to document the health impact, feasibility and safety of the outpatient/at-home ASCT models, which are increasingly being applied around the world.

## Introduction

Hospital-at-home involves the provision of clinical care in the patient’s home, their natural environment, rather than in the hospital. The reasons for this paradigm shift, which we are already witnessing, lie in different factors such as the increase in the consumption of health services, their costs, and the rationalization of the use of hospital beds (1). The evidence is limited by the few controlled and randomized trials (2–4), by the difficulty in patient selection, or by the small sample size. Most studies show that this type of care is feasible and effective, which is confirmed in a meta-analysis (5) on home hospitalization programs.

Ambulatory ASCT model was recognized for its positive results in terms of effectiveness and safety since the end of the last century (6), reducing length of stay, nosocomial infections, costs, and generating greater comfort for patients. However, this model also shows associated risks (3, 7–10).

With the exponential increase in autografts performed in developed countries, and the consequent increase in care costs, the optimization of ambulatory care provision was established as a strategic objective on the basis of the results of health-care and quality of life of patients (11–13). However, these models are still not routine procedures for different reasons, which the Italian Group for Stem-Cell Transplantation (GITMO) (14) have analyzed, highlighting three essential elements: the identification of the selection criteria for candidates for ambulatory ASCT, the definition of a standard procedure, and the characterization of the criteria for hospital admission during the aplastic phase.

Likewise, the advent of CAR-T platforms, with an increased range of indications and more limited toxicity, has increased interest in early patient discharge in the ambulatory setting while maintaining close patient monitoring. It appears that the future of CAR-T therapy is outpatient management with a careful planning to optimize patient safety.

The review of home hospitalization focused on ASCT, presented below, aims to highlight the potential impact of the model in optimizing care delivery. And this in terms of health results, in aspects such as the perception of the user and caregiver, economic costs or in the use of new technologies, in contrast to conventional and exclusive hospital management.

## Methodology

A systematic review to document the health impact, feasibility and safety of the ambulatory ASCT models, which are increasingly being applied around the world, was conducted.

### Data sources and searches

PubMed is used as a search engine to identify relevant and quality bibliography. Published articles in the PubMed database as at February 1, 2024 were searched by two investigators, using a search strategy developed for PubMed that included a combination of appropriate keywords, MeSH (Medical Subject Headings) and non- MeSH terms. No language or time restrictions were applied.

The descriptors used were autologous stem-cell transplantation, hospital at home, outpatient transplantation, at-home transplantation, and ambulatory transplantation. And “and” was used as a Boolean operator. Additional S1 File illustrates the search strategy details. An effort to identify additional eligible studies was made by reviewing the references of the included studies. The full text of the selected articles was accessed through different means. The PRISMA (Preferred Reporting Items for Systematic Reviews and Meta-Analyses) was used to improve transparency in our findings. The PRISMA checklist is available in S2 File.

### Inclusion and exclusion criteria

We looked for home hospitalization studies focused on ASCT, to highlight the possible impact of the model in optimizing health results, in contrast to conventional hospital management. Therefore, we sought models of care that aimed to avoid or reduce hospitalization duration and pressures on healthcare services. Studies carried out on the pediatric population were excluded, as well as duplicates articles, irrelevant studies to autologous stem-cell transplantation or without comparison between outpatient and inpatient settings. The PRISMA Flow Diagram is available in S3 File.

### Study selection: data collection process and data items

We then proceeded to critically read each and every one of the selected publications, which are reflected later, as they are original articles that show the experience of researchers in the area of ASCT, in diseases basically onco-hematological with non-hospital models.

Two authors screened titles and when the information provided in the title was inconclusive, the abstract was consulted for eligibility, followed by full-text review to be considered.

Details pertaining to author, publication year, country, study characteristics and design, study sample, outpatient interventions scheme, and reported outcomes were extracted using a spreadsheet software, Microsoft Excel, because Excel is a powerful tool for data analysis, as it allows users to manipulate and analyze large amounts of data quickly and easily.

## Results

### Feasibility of a hospital-at-home program for ASCT

Currently, home hospitalization presents a great diversity of care models (1). It entails a modality of health care capable of carrying out diagnostic and therapeutic procedures and care at home similar to those provided in hospitals. Its characteristics are set out in **Table 1**.

**Table 1:** Characteristics of hospitalization at home units.

Defining characteristics of home hospitalization
The patient is treated at home for an acute illness that requires hospitalization
Treatment requires hospital-grade technology.
The health system assumes that these patients remain dependent on the hospital
Drug administration, complementary tests and the rest of the services will be carried out with the same speed as that applied to admitted patients.
Home health care services have extensive hourly coverage and 24-hour continuity of care.
Care is carried out in a coordinated manner and is similar to that of the admitted patient.
This action requires the informed consent of the patients.
Its purpose is to reduce hospital stays, either by avoiding admission or by shortening it (early discharge pattern).

Ambulatory ASCT programs have been favored by the improvement of antimicrobial prophylaxis and therapy, the prevention of mucositis, technological advances, adequate infrastructure and the existence of an experienced multidisciplinary team, among other aspects. The selected patients share requirements such as living in a geographical area less than 60 minutes distant from the hospital, suffering from a disease whose evolution is in a phase that fully justifies admission to the hospital, having a person who performs the functions of caregiver, and, finally, to have the consent of the patient and their families.

The establishment of an ambulatory ASCT program improves health care for patients who require this procedure in the natural history of their hematological process (10, 12, 13).

Most autologous transplants are performed for hematological malignancies; specifically, they are the standard in the management of multiple myeloma and also have a well-established role in the treatment of Hodgkin and non-Hodgkin lymphomas. The advantages of home hospitalization are evident from several points of view (7–10):

✔ For the patient: personalized assistance in their family environment, better perception of patient care, greater ability to carry out activities of daily living. In short, high patient satisfaction.

✔ For the family: unnecessary trips are avoided, greater comfort is reached.

✔ For the hospital: resources are optimized by saving hospital stays, lower risk of nosocomial infections, fewer clinical complications such as confusional and agitation disorders, etc. Ultimately, it reduces healthcare pressure and the waiting list for ASCT. On the other hand, this innovative activity gives “prestige” to the hospital.

✔ For healthcare managers: it clearly represents economic savings in healthcare, mainly due to the reduction in hospitalization and greater patient-bed rotation, without there being any difference in mortality and readmission rates.

### Ambulatory ASCT models

Various studies have evaluated the safety, effectiveness, and feasibility of ambulatory ASCT, in its different models, as an optimal approach to the management of hospital stays, with the consequent potential savings in healthcare resources and costs (**Table 2**).

**Table 2:** Ambulatory ASCT models.

Early discharge model							
Author	CVC. Q	STI	MAP	No. patients	Readmissions (%)	Median (days) duration of readmission	TRM (%)
**Ferrara** (15)	Inpatient clinic	Inpatient clinic	Outpatient clinic	28	35.7	10	0
**Martino** (16)				382	18.8	4	1
**Faucher** (17)				131	86.6	9	1.5
**Paul** (18)				82	67	9	0
**Abid** (19)				10	40	6.9	0
Delayed admission model							
**Anastasia** (20)	Outpatient clinic	Outpatient clinic	Inpatient clinic	123	6	10	0
Total outpatient model							
**Gertz** (21)	Outpatient clinic	Outpatient clinic	Outpatient clinic	716	58 (<65 years) 71 (≥ 65 years)	3 (<65 years) 7 (≥ 65 years)	1.1 (global)
**Gertz** (22)				1078	25	≥5	0.3
**Kassar** (23)				90T*	58	2.2	0
**Holbro** (24)				91	84	8	0
**Shah** (25)				377	55	6	0.3
**Kodad** (26)				724	33.8	6	0.4
**Yip** (27)					100	6	
Mixed inpatient-outpatient model							
**Morabito** (28)	Outpatient clinic	Inpatient clinic (day 0 to 2)	Outpatient clinic	29	43	9	
**Clemmons** (29)				18	0	0	
At-Home model							
**Martino** (30)	Inpatient clinic	Inpatient clinic	Home	15	15		0
**Fernández Avilés** (9)				50	8	8	
**González-Barrera** (31)				84	12		0

CVC, central venous catheter insertion; Q, high-dose chemotherapy administration; STI, stem-cell infusion; MAP, management of aplastic phase; TRM, transplant-related mortality; T*, transplants. Martino et al. Multiple Myeloma Outpatient Transplant Program in the Era of Novel Agents: State-of-the-Art. Front Oncol. 2020; 10:592487.

Different publications are shown on the different existing models, with the patients included in each case and the main clinical and health results that determine hospital bed resource they consume.

### Patient selection

Patient selection is the key to achieving successful ambulatory ASCT. The inclusion and exclusion criteria used in studies published over recent decades reflect common aspects, although they show differences in their values ​​and in the number of conditions required (10, 13, 14, 17) (**Table 3**).

**Table 3:** Inclusion criteria for ambulatory ASCT.

Inclusion criteria
PATIENT
Age ≤ 65 years
Good general health condition, ECOG ≤ 2
Normal cardiac, lung, liver and renal function
Recent documented infection with a proven secondary prophylaxis
Absence of refractoriness to platelet transfusion
Signed written informed consent
TRANSPLANT CENTER
Outpatient clinics available 24 h per day or bed reserved in the transplant unit
Dedicated phone line 24h × 365 days to allow patients or their caregivers to contact an expert physician of the transplant team
DISEASE
CR or PR before the ASCT
No symptomatic advanced disease
CAREGIVER
Availability of a suitable caregiver 24 h per day, 7 days a week
HOME
Clean house
Travel time from home to the hospital less than 60 min at rush hours

ECOG, Eastern Cooperative Oncology Group; CR, complete remission; PR, partial remission or partial response; ASCT, autologous stem-cell transplantation. At-Home HSCT. Francesc Fernández-Avilés and Gonzalo Gutiérrez-García. https://www.ebmt.org/sites/default/files/2019–01/2019_Book_TheEBMTHandbook.pdf.

### Incidence of complications

The incidence of the most common complications in patients treated in a home environment varies widely depending on the series. Fever is, by far, the most common complication. Febrile neutropenia is the most frequent cause of hospital readmission in ambulatory care programs for patients treated with ASCT. It shows a wide range, between 20–86%, although lower percentages respond to the use of parenteral antibacterial prophylaxis. A recent study showed that the use of piperacillin/tazobactam and fluoroquinolone prophylaxis may effectively prevent episodes of neutropenic fever and hospitalizations in lymphoma patients managed in our at-home ASCT care model (32). However, it is possible that, sometimes, the febrile episodes may have a non-infectious etiology, and engraftment syndrome could play a more significant role. The study by *Rodríguez-Lobato et al.* (33) found that, for patients with multiple myeloma in at-home ASCT, the avoidance of G-CSF and the addition of primary prophylaxis with corticosteroids after ASCT minimizes the incidence rates of neutropenic fever and engraftment syndrome.

Mucositis varies between 6–100% (some authors focus on grade 2–3 mucositis, while others also include grade 1 mucositis), while gastrointestinal disorders range between 17–53%. The values ​​of the different series are shown in **Table 4**.

**Table 4:** Incidence (%) of the main complications in ambulatory ASCT.

First author	Febrile neutropenia	Mucositis	Nausea-vomiting	Diarrhea
Jagannath (1997) (6)	50	31		
Hermann (1999) (34)	61	35		53
Morabito (2002) (28)	50	56		
Fernández Avilés (2006) (9)	76	24		35
Anastasia (2009) (20)	76	100		
Holbro (2013) (24)	78	6		
Martino (2015) (30)	50	80		
Graff (2015) (35)	29.5	6		14
Paul (2015) (18)	22			
Lisenko (2017) (36)	57	100		
Shah (2017) (25)	36		41	41
Abid (2017) (19)	40			
Obiozor (2017) (37)	20	7	17	17
Kodad (2019) (26)	74.4	31.8		31.4
Larsen (2022) (38)	52		34	34
González-Barrera (2023) (31)	86	44		
Al-Anazi (2023) (39)	32	8		

### Causes of hospital readmissions

Hospital readmission is a frequent occurrence in patients with ASCT on an ambulatory model. It happens in a very wide range, which oscillates between 8–90% due to the disparity and laxity of criteria recorded in the different published series. The causes are very varied, highlighting the patient’s or caregiver’s own will or claudication, hemodynamic instability, persistent fever, cardiorespiratory disorders, altered level of consciousness, uncontrolled nausea/vomiting or diarrhea, or mucositis oral requiring total parenteral nutrition and opioid analgesics, among other reasons.

### Quality of life

The general belief that patients treated on an ambulatory basis perceive greater physical and psychological well-being is controversial. A similar pattern in quality of life is observed in both outpatients and inpatients, worsening between days 4–6 post-transplant and improving on day 12–16, when in most cases recovery of neutrophil values occurs, and extramedullary toxicity is overcome.

The few studies that provide data on the quality of life of the patient treated in an outpatient/home ASCT model have important limitations; they are observational, non-randomized studies, in which the patient chooses the transplant modality, which makes the results often inconsistent, contradictory, and influenced by the initial choice, or not finding statistically significant differences with respect to the conventional model (9, 13, 40, 41).

*Summers et al.* (42) found that emotional, social and physical well-being, as well as quality of life, were significantly better in ambulatory than in hospitalized patients. *Fernández Avilés et al.* (9) used an anonymous questionnaire after completing the procedure. All patients and caregivers felt safe at home and almost all indicated that they would choose the home model again and recommend it to another patient.

On the other hand, *Martino et al.* (40) confirmed that global quality of life was not significantly different between ambulatory and hospitalized patients during the transplant period. In conclusion, the ambulatory ASCT model did not improve or affect the patient’s overall quality of life compared to the hospital standard.

The use of telemedicine in the Hematopoietic Transplant environment allows equitable and universal healthcare, accessible to the most disadvantaged sectors or from geographical areas that are difficult to access. They can positively affect the physical and psychological state of patients and, therefore, their overall quality of life. But the clinical experience of telemedicine in this area is very limited. The pilot study carried out by *Nawas et al.* (43) found a high rate of satisfaction among patients, but not among the responsible clinician, due to technological barriers that caused delays and a suboptimal physical examination as the main causes of their dissatisfaction. The study by *Mussetti et al.* (44), in Hospitalet (Spain), with transplant patients during the COVID19 pandemic, confirmed the obvious advantages for doctors and patients that these technologies provided, although technological problems still represent a challenge, especially for patients who live alone or without caregivers. *González Sierra et al.* (31), at the University Hospital of Granada, uses a mixed post-transplant care model: the patient goes to the outpatient clinic 2–3 days a week for analytical monitoring and physical examination, and on the rest of the days they have a mobile application that ensures direct daily doctor-patient communication.

The use of this technology is a complementary tool in the management of hematopoietic transplantation that can improve the physical and psychological state of both patients and their caregivers, but it must be adapted to their capabilities to avoid technological limitations.

### Impact of caregivers on home ASCT results

Most hospitals offering ambulatory transplantation require “twenty-four seven” availability of a caregiver, at least for the duration of the aplastic phase. Caregivers play a crucial role in the success of the ambulatory procedure, spending most of their time with the patient, affecting them in multiple facets of their life, and carrying a very significant physical and emotional burden. The profile of the caregiver would be the patient’s couple, an active worker in half of the cases, with a high academic level, female, and aged around 54 years (45).

Several trials showed better overall survival in patients with availability of a caregiver compared to those undergoing ASCT without such support. Likewise, the decrease in readmissions, with the consequent reduction in costs, and the feasibility associated with the ambulatory approach seem to be mediated mainly by the effort of the caregivers. Furthermore, the lack of a caregiver was the most frequent cause of patient refusal to participate in an outpatient/at-home program (40, 42, 46, 47).

Therefore, rigorous studies are needed to evaluate better the emotional, economic-labor burden and quality of life of caregivers, their impact on the results and quality of life of patients undergoing an ambulatory or home transplant.

### Costs and impact on health services

Numerous studies have indicated that ambulatory ASCT is cost-effective, mainly due to the shorter length of hospital stay, which ranges between 3 and 17 days (5–7, 9, 10, 12, 13, 17, 19, 29–31). It offers specialized medical care to patients in their homes for diseases that normally require hospitalization (13, 41). All articles that compare costs between ambulatory and hospital care confirm a reduction in favor of the ambulatory model, ranging between 19.32% and 53% (**Table 5**). However, the limitations of these publications are important. Therefore, selection criteria for hospitalization of ambulatory patients, which differ between studies, and the variability of the models, may make the comparison of the results very complicated. Only one randomized clinical trial was found in our review, *Faucher´s one* (17). There is a lack of studies with an appropriate design that evaluate the real savings obtained with outpatient/home ASCT programs. It is known that more than half of the total costs billed by autologous transplant correspond to expenses for hospital admissions, which has a significant effect on health systems. However, other factors involved in the expense should be considered, such as the outpatient/home model used, with or without a bed freed up during out-of-hospital follow-up, the hospital readmission rate of each program, the different existing health and economic models or other relevant expenses (drugs, laboratory, healthcare personnel fees, sick leave).

**Table 5:** Cost reduction of the ambulatory *vs.* hospital model in ASCT.

Author	Cost reduction percentage
Jagannath (1997) (6)	26.40
Meisenberg (1998) (7)	34.32
Frey (2002) (47)	28.37
Fernández Avilés (2006) (9)	46.48
Faucher (2012) (17)	19.32
Holbro (2013) (24)	31.36
Abid (2017) (19)	32.72
Shah (2017) (25)	29.70
Martino (2018) (38)	42.34
Clemmons (2017) (29)	53
Dunavin (2020) (48)	43.15

Taken from González MJ et al. (13) and completed after reviewing the literature.

### Worldwide distribution of published clinical studies of home ASCT

Although different researchers (10, 13, 41, 49) have focused on studying the impact of the ambulatory model in stem-cell transplantation, the overall comparative effects have not yet been systematically evaluated. **Table 6** shows the publications from different continents that analyze the evidence on the effects of ambulatory treatment, in terms of health results, costs and experience in care.

**Table 6:** Worldwide distribution of ambulatory ASCT for hematological malignancies.

Country First author	Design study	Fever (%)	Muc (%)	READM	Length of stay	MRT	Quality of life	Cost reduction
AMERICA								
1. USA								
**Jagannath** (6)	CC, M	42.3	26.2	19 (21%)	9/15			13.172$
**Meisenberg** (50)	CC, U			40 (29.6%)	2.81/ 18.33	1 0.7%		
**Meisenberg** (7)	CC, U	64.3	14.3	14 (18.8%)	2.7/ 17.3		+, NM	10.300$
**Frey** (47)	P, U	5	24	12 (57.1%)	2/18	0	S, M	11.775$
**Stiff** (46)	CC, U	28	100	28 (28%)	6/21	4 4%	S, M	16.000$
**Kassar** (23)	R	49	18	40 (45%)	3.7	0		
**Gertz** (21)	P, no AL			438 (61.1%)	4	8 1%		↓ NQ
**Graff** (35)	R, U	29.5	6.3	47 (49%)	5.4/ 19.2	0 1 year		40.000$
**Paul** (18)	CC, R, U	58.53		55 (67%)	9/18	0	+, NM	NQ
**Reid** (51)	R, U	52	16	1 (1.72%)	12/18	0	+, NM	17.000 $
**Shah** (25)	R, U	40.8	4	207 (55%)	6/11	1 0.3%	+, NM	124.000$
**Obiozor** (35)	R, U, 3 arms	19.29	7	57 (32.5%)	7/13	0		
**Clemmons** (29)	R, U			0	0			2.209 $
**Dunavin** (48)	RC, M			107 (55%)	6/22	2 1%		35.544$
**Larsen** (38)	R, U	52	4	158 (31.6%)	8/15			
**Sung** (52)	CC, U	64.7		21%*	4.6/ 3.7	0 1 year	+, M	
2. Canada								
**Leger** (53)	R, U	56.2	29.7	54 (90%)	7/	1 1.7%	+, M	NQ
**Summers** (42)	O, M, P	67	94	19 (90.5%)	7/14		+, M	Similar **
**McDiarmid** (54)	CC, U	25		122 (24%)	2/14	23 4.7%	+, NM	NQ
**Holbro** (24)	R, U	78	5.5	76 (84%)	8/28	0		19. 522 $
**Kodad** (26)	R, U	74.4	31.8	245 (33.8%)	6/	3 0.4%		
3. Australia								
**Jaime-Pérez** (55)	R, U	46.1	25.5	23 (22.5%)	5/	5.5% 1 year		
**Herrmann** (34)	CC, U	60.8	0	18 (35.3%)	3/	2 3.9%		
**Tan** (56)	R, U	73.7	35	71 (52.6%)	9/19	3 2.2%		13.000 to 16.000 $
EUROPE								
1. Netherlands								
**Westermann** (57)	P, no AL, 3 arms	87		46 (71.8%)	1.5/6/14		+, M	
2. Italy								
**Morabito** (28)	CC, U	25		12 (43%)	9/20			
**Ferrara** (15)	P	28.5	7	10 (35.7%)	10/	0		
**Anastasia** (20)	R, U	76		5 (6%) early READM	10	0		NQ
**Ferrara** (58)	P, U, 2 arms G-CSF vs PEG	30.4	24.4	PEG:6 (12%). G-CSF:30 (26%)	4/9	1 0.6%		
**Martino** (11)	P, no AL			2 (18.1%)	4/18	0		
**Martino** (16)	R, M	30.8	9.6	98 (18.8%)	4/	4 1%		NQ
**Martino** (30)	P, no AL, 3 arms	28 40	4 0	2 (8%) 2 (13%)	4/19 4/19	0	+, NM	
**Martino** (40)	P, O	35.9	18	2 (3.1%)		0	S, M	
3. France								
**Faucher** (17)	AL, M	57		57 (86.6%)	9/12	1 1.5%		2.015€
4. Spain								
**Fernández-Avilés** (9)	CC, U	76	24	4 (8%)	8/25	0	+, M	2.905€
**G-Barrera** (31)	R, U	86	44	10 (12%)		0	+, NM	
5. UK								
**Yip** (27)	R, U	43	6	54 (100%)	6/		+, NM	NQ
6. Germany								
**Lisenko** (36)	R, U	67	19	7 (33.3%)	5	0		
7. Poland								
**Dytfeld** (59)	P, U	0	20	1 (20%)		0	M, NM	24.962€
ASIA								
1. Singapore								
**Abid** (19)	CC, U	40		4 (40%)	6.9/ 18.3	0		7.597$
2. Saudí Arabia								
**Al-Anazi** (39)	R, U	32	8	34 (43%)		0		NQ

ASCT, autologous stem-cell transplantation; PEG, pegfilgrastim; G-CSF, granulocyte colony-stimulating factor (filgrastim); Country/First author, distribution of publications by continents and countries, reflecting the first author and bibliographic citation. Design study: CC, case control; M, multicenter; O, observational; U, unicentric; P, prospective; R, retrospective; RC, retrospective cohort; AL, randomized. Model: A, ambulatory (outpatient clinic); D, domiciliary (at home). Fever: percentage of ambulatory patients who have a temperature >38,2°. Muc.: mucositis grade >2. READM: (Readmissions), are expressed in absolute and relative frequency, except * which is expressed in average hospitalization time. Length of stay indicates the number of days of hospitalization in the ambulatory model. The length of stay in the hospital model is expressed below in case of comparison of models. MRT: treatment-related mortality at 100 days, in absolute number and percentage. Quality of life: better quality of life (+), either through validated measurement scales, surveys or questionnaires (M), or, in other cases, not measured (NM) and based on opinions of patients/caregivers and/or authors. In other series, no difference was recorded between the home and hospital groups (S: similar). Cost reduction: the average savings per patient is expressed in dollars (from the US, Canada, or Australia, depending on the origin of the publication) or euros. In some series, cost reduction (↓) in ambulatory models is assumed due to a reduction in hospital stay, but it is not quantified (NQ). **The Summers series shows similar costs in the two models, but they do not compute the days of hospitalization.

↓errata. Valid: NC.

It is worth highlighting certain limitations of the studies that are set out in **Table 6** and which we briefly summarize: 1. Health outcome data show wide variability between the different series. 2. Regarding quality of life, only some studies use validated measurement scales or satisfaction questionnaires. 3. To evaluate costs, it must be taken into account that the financing of health systems is highly variable in different countries. 4. It must also be reflected that the savings recorded in most studies are based only on the reduction in hospital length of stay while other economic aspects of the procedure are not included. 5. The designs of the studies are heterogeneous, with many being retrospective, without a comparison group; others are prospective, with several arms, but are not randomized; few have a case-control design and sufficient sample size, and we only have one randomized, multicenter clinical trial, therefore with greater methodological rigor.

## Discussion

Several studies have confirmed the feasibility and safety of ambulatory ASCT, reinforcing it as an alternative to standard hospitalization. Its potential benefits in terms of health outcomes and quality of life for the patient, or savings of healthcare resources for organizations, are obvious.

The systematic review and meta-analysis by *Owattanapanich et al.* (10), which included nine studies with a rigorous design, showed a significantly lower probability of developing febrile neutropenia and septicemia in patients undergoing ambulatory management compared to those who received an ASCT as inpatients, with the Ib level of evidence. In addition, it reported lower costs and higher levels of patient satisfaction in that group. We also have a single, randomized, multicenter clinical trial that found a shorter hospital stay, as well as a lower cost of the procedure in the group of home patients (17) (evidence Ib).

These data should facilitate broader implementation of the ambulatory strategy. However, it has not yet been established as a routine procedure, and many hospital centers are reluctant to adopt this approach, probably due to lack of adequate infrastructure.

Over the last decades, other studies have also shown that the home model improves the quality perceived by the patient and their families, reduces the duration of hospitalization, the waiting list for hematopoietic transplant, and minimizes exposure to nosocomial microorganisms. In short, it optimizes healthcare resources and clearly represents financial savings in healthcare, without any difference in mortality rates with respect to the hospital model. However, many of them are non-prospective and non-randomized studies and, therefore, this methodology limits the validity of the results, with level III evidence.

In our systematic review, most of the patient populations undergoing transplantation suffered from multiple myeloma. Generally, high-dose melphalan conditioning (140–200 mg/m^2^), administered in one or two consecutive days, was chosen as cytoreductive regimen in these patients and may be more suitable for a fully outpatient management. The second patient population is Hodgkin and non-Hodgkin lymphomas receiving BEAM as conditioning. BEAM is administered for six consecutive days, even with two components given every 12 hours, making it difficult to administer on an ambulatory basis.

On the other hand, the incidence of early adverse events, such as neutropenic fever and oral/gastrointestinal mucositis, was much lower in patients with multiple myeloma who received an ASCT.

Due to the ease of administration of high-dose melphalan and the relatively low extra-hematological toxicity, patients with multiple myeloma are ideal candidates for ambulatory ASCT.

The current systematic review found positive results among patients underwent an ambulatory ASCT than among those who had an inpatient ASCT. This could be an appealing reason to utilize the first strategy in addition to its reported better cost-effectiveness, and higher patient satisfaction levels. However, given the methodological heterogeneity of existing publications, and the limited validity of the observational nature of the included studies, future controlled and randomized studies are still needed to confirm the potential benefit of the ambulatory procedure, in terms of health outcomes, cost-effectiveness, or quality of life. In relation to the cost studying, other factors involved in the expense should be considered in addition to the clinical aspects, such as the outpatient/home model used, the different existing health and economic models, or other expenses (role of the caregivers, drugs, laboratory, healthcare personnel fees…).

## Conclusions

The ambulatory ASCT is safe, feasible, and cost-effective, even in patients over 65 years of age and with certain non-limiting comorbidities, especially in work groups with consolidated outpatient projects. So, it seems to us that we are in a position to propose certain inclusion criteria to be candidates for ambulatory ASCT, especially for teams that want to start an outpatient ASCT program: multiple myeloma patients, age less than or equal to 65 years, good performance status (ECOG ≤ 2), favorable comorbidity profile (normal cardiac, lung, liver and renal function), availability of a suitable and full-time empowered caregiver and travel time from home to the hospital less than 60 min at rush hours. The main advantages reported of performing this ambulatory procedure include saving beds, reducing hospital costs, and lowering the rates of infections. These aspects should be considered by healthcare providers. Due to the difficulty to carry out randomized and controlled trials in this setting, specific and rigorous inclusion and exclusion selection criteria are required for the routine use of the ambulatory ASCT model.

## Data availability statement

The original contributions presented in the study are included in the article/**Supplementary Material**. Further inquiries can be directed to the corresponding author.

## Author contributions

MP: Writing – review & editing, Writing – original draft, Visualization, Software, Methodology, Investigation, Formal analysis. FF: Writing – review & editing, Visualization, Supervision, Project administration, Methodology, Investigation, Formal analysis, Conceptualization.
