# Supplementary material for: Ambulatory models for autologous stem-cell transplantation: a systematic review of the health impact
Source: Front Immunol. 2024 Jul 16;15:1419186. doi: 10.3389/fimmu.2024.1419186 (PMC11287121; doi:10.3389/fimmu.2024.1419186)
Supplement: Supplementary file 1 [file DataSheet_1.docx]

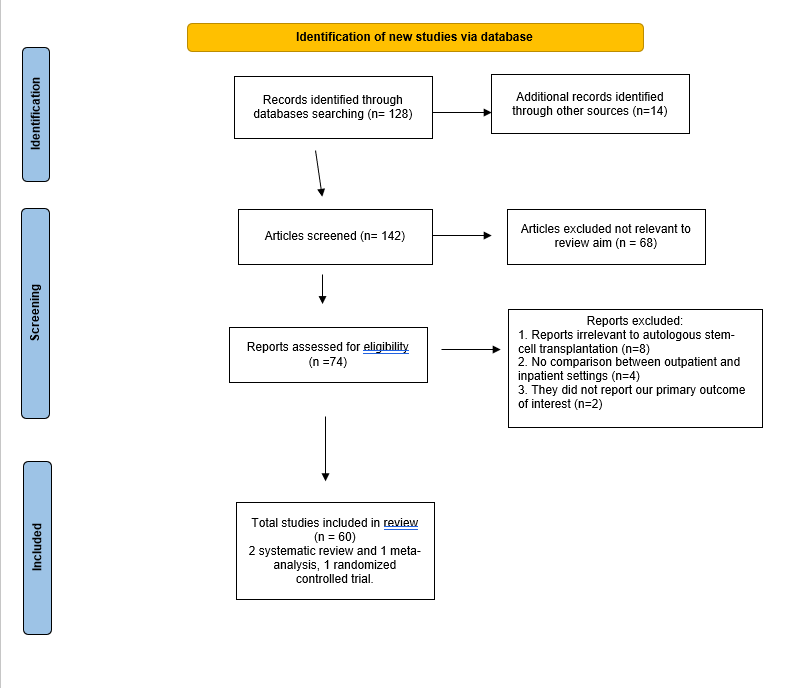


*From:*  Page MJ, McKenzie JE, Bossuyt PM, Boutron I, Hoffmann TC, Mulrow CD, et al. The PRISMA 2020 statement: an updated guideline for reporting systematic reviews. BMJ 2021;372:n71. doi: 10.1136/bmj.n71
