# Supplementary material for: Ambulatory models for autologous stem-cell transplantation: a systematic review of the health impact
Source: Front Immunol. 2024 Jul 16;15:1419186. doi: 10.3389/fimmu.2024.1419186 (PMC11287121; doi:10.3389/fimmu.2024.1419186)
Supplement: Supplementary file 2 [file DataSheet_2.docx]

**URL:**

https://pubmed.ncbi.nlm.nih.gov/?term=%28%22Transplantation%2C+Autologous%22%5BMesh%5D%29+AND+%22Outpatients%22%5BMesh%5D&sort=date

https://pubmed.ncbi.nlm.nih.gov/?term=autologous+stem+cell+transplantation+and+outpatient+%5BTI%5D

https://pubmed.ncbi.nlm.nih.gov/?term=%28%22Ambulatory+Care+%22%5BMesh%5D%29+AND+%22Transplantation%2C+Autologous%22%5BMesh%5D

https://pubmed.ncbi.nlm.nih.gov/?term=autologous+stem+cell+transplantation+and+quality+of+life+and+outpatient

https://pubmed.ncbi.nlm.nih.gov/?term=autologous+stem+cell+transplantation+and+ambulatory

https://pubmed.ncbi.nlm.nih.gov/?term=autologous+stem+cell+transplantation+and+hospital+at+home

https://pubmed.ncbi.nlm.nih.gov/?term=%28%22Transplantation%2C+Autologous%22%5BMesh%5D%29+AND+%22Ambulatory+Care%22%5BMesh%5D&sort=date
